# Wound Myiasis in a Patient with Squamous Cell Carcinoma

**DOI:** 10.1100/tsw.2009.138

**Published:** 2009-11-01

**Authors:** Mohammad Namazi, Mohammad Kazem M. K. Fallahzadeh

**Affiliations:** ^1^ Dermatology Department, Faghihi Hospital, Shiraz, Iran; ^2^ Medicinal and Natural Products Chemistry Research Center and Dermatology Department, Shiraz University of Medical Sciences, Shiraz, Iran

**Keywords:** myiasis, wound, treatment

## Abstract

A 60-year-old, otherwise healthy, male farmer presented to our Dermatology Department with a large ulcer on his lower right leg. The lesion had started as a small papule 6 months before, which became eroded and transformed into a rather rapidly progressive ulcer. On careful inspection, numerous larvae were found moving within the wound. The larvae were analyzed and found to be Lucilia sericata (the green bottle blowfly). The lesion was diagnosed histopathologically as squamous cell carcinoma. The myiasis was treated by submerging the wound in a dilute permanganate potassium solution.

